# Effects of Chlorophyll *a* and *b* in Reducing Genotoxicity of 2-Amino-3,8-dimethylimidazo[4,5-F]quinoxaline (MeIQx)

**DOI:** 10.3390/biology11040602

**Published:** 2022-04-15

**Authors:** Serap Kocaoğlu Cenkci, Bülent Kaya

**Affiliations:** 1 Department of Nutrition and Dietetics, Faculty of Health Sciences, Akdeniz University, Antalya 07058, Turkey; 2 Department of Biology, Faculty of Science, Akdeniz University, Antalya 07058, Turkey; bkaya@akdeniz.edu.tr

**Keywords:** chlorophyll, MeIQx, antigenotoxicity, *Drosophila melanogaster*, SMART

## Abstract

**Simple Summary:**

We aim to determine whether chlorophyll *a* and chlorophyll *b* pigments in green vegetables and fruits have antigenotoxic effects against the MeIQx compound, which is frequently formed during the cooking of meat and meat products.

**Abstract:**

In this study, the protective effects of chlorophyll *a* and chlorophyll b (0.5 and 1 µM) against the heterocyclic amine compound 2-amino-3,8-dimethylimidazo[4,5-f]quinoxaline (MeIQx, 4.69 µM, 9.38 µM, 23.45 µM) with somatic mutation and recombination test in *Drosophila melanogaster* are investigated. Chronic applications are performed to transheterozygous larvae with respect to two recessive genes, *mwh* (multiple wing hair) and *flr^3^* (flare), by using *Drosophila* strains. The genotoxic effects of MeIQx are primarily determined for third instars larvae. In antigenotoxicity studies, two different application groups are constituted. While for the first group doses of chlorophyll *a*, *b*, and MeIQx are given to the third instars larvae simultaneously, for the second group doses of MeIQx are applied at the third instars after doses of chlorophyll *a* and *b* are given to at the second instars larvae. Chlorophyll *a* and *b* are effective in reducing genotoxic effects of MeIQx by both applications on individuals and it is observed that the pretreatment method is much more effective than the simultaneous one. There are similar results for chlorophyll *a* and *b* in efficacy.

## 1. Introduction

Nutrition is an indispensable requirement of life which affects health and quality of life. Nutrition habits vary between societies. In some societies, a vegetable-based diet is preferred, while in others meat and meat products are preferred more. Various preparation methods have been developed to make foods more edible from past to present. These methods not only make the foods appetizing in terms of taste and appearance, but also delay their deterioration during the storage process and provide more reliable foods from a bacteriological point of view [1]. However, many genotoxic substances and cooking toxicants are formed during the processing and cooking of foodstuffs. Heterocyclic amine (HCA) compounds, which are not found in raw meat but are formed by cooking meat and meat products at high temperatures, can be given as examples. Raw meat and fish muscle contain all necessary precursors for HCA formation. Heterocyclic amines are small molecules formed by creatine/creatinine, amino acids and/or sugar at high temperatures [2]. As the cooking temperature increases, the number of molecules formed also increases. In addition, the cooking technique also affects the amount of substance formed. Boiling produces the least amount of HCA, while techniques that require higher temperatures, such as deep frying, baking and grilling, result in higher and variable amounts of substance [3].

In addition to the intake of harmful food components by nutrition, beneficial food components are also taken. Vegetables and fruits are not only sources of protein and carbohydrates, but also contain many different functional chemical compounds. Green and yellow pigmented vegetables contain very valuable but low concentrated compounds such as vitamins. Studies have shown that there are a large number of substances with antioxidant and antimutagenic effects [4,5]. Many epidemiological and experimental studies have further shown that antioxidant-rich foods reduce certain cancer types and mutation frequency [6,7,8,9,10,11].

Mammalian model organisms play a central role in determining the genetic risks to which the human population is exposed. However, some ethical limitations in the use of experimental animals have led to the development of different alternative methods. *Drosophila melanogaster* is a eukaryotic organism with a suitable suborganization that can replace mammals. These flies are advantageous experimental organisms for genetic studies. Many studies on human diseases such as cancer, neurological diseases, metabolism disorders, cardiovascular diseases and malformation disorders support the use of *Drosophila melanogaster* [12,13,14]. The most commonly used short-term mutation test to detect genetic changes in *Drosophila* somatic tissues is the wing somatic mutation and recombination test (SMART). The *Drosophila* SMART is a versatile, effective and inexpensive short-term in vivo genotoxicity test for determining the genotoxicity of single compounds or mixtures.

The main objective of this study is to determine whether chlorophyll *a* and chlorophyll *b* pigments in green vegetables and fruits have antigenotoxic effects against the genotoxic effect of the MeIQx compound, which is frequently formed during the cooking of meat and meat products, using *Drosophila* SMART. We gave chlorophyll pigments and HCA compound to flies in two different application times. In the first group, flies were simultaneously exposed to chlorophyll and HCA compound at the larval stage for 72 ± 4 h. In the second group, flies were exposed to chlorophyll pigments earlier in the form of pre-application for 48 ± 4 h at the larval stage and exposed to HCA for 72 ± 4 h at the larval stage. Thus, we planned to determine the time interval in which chlorophyll pigments, which are thought to have potential antigenotoxicity, are more effective in reducing genotoxicity of MeIQx.

## 2. Materials and Methods

### 2.1. Chemicals

2-Amino-3,8-dimethylimidazo[4,5-f]quinoxaline (MeIQx, C_11_H_11_N_5_, CAS No. 77500-04-0) was purchased from Toronto Research Chemicals; chlorophyll *a* (C_55_H_72_N_4_O5_Mg_, CAS No. 479-61-8), chlorophyll *b* (C_55_H_70_MgN_4_O_6_, CAS No. 519-62-0), dimethyl sulfoxide (DMSO, CAS No. 67-68-5), ethyl methane sulfonate (EMS; CAS No. 62-50-0) were purchased from Sigma. Chlorophyll *a* and *b* were dissolved in DMSO at 0.5% and diluted in distilled water to obtain the test concentrations (0.5, and 1 µM). Mutagen was dissolved in DMSO at 0.5% and diluted in distilled water to obtain the test concentrations (4.69, 9.38 and 23.45 µM).

### 2.2. Drosophila Strains

Two *Drosophila melanogaster* strains were used: the multiple wing hairs strain with the genetic constitution *mwh/mwh*, and the *flare-3* strain with the genetic constitution *flr^3^/In (3LR) TM3, Bd^S^*. More detailed information on the genetic symbols and descriptions can be found in [15].

### 2.3. Experimental Procedure

The wing spot test is based on the loss of heterozygosity in somatic cells of larvae [16]. The transheterozygous larvae were obtained by parental crosses between *flr-3* virgin females and *mwh* males. Eggs from this cross were collected during 8 h periods in culture bottles containing standard growth medium. At the application time, the larvae were floated off with tap water and then transferred into plastic vials containing 1.5 g dry *Drosophila* Instant Medium (Carolina Biological Supply Company Burlington, NC, USA) rehydrated with 9 mL of the freshly prepared test solutions. The larvae were fed with different concentrations of the test compound, and feeding ended with pupation of the surviving larvae. All experiments were performed at 25 ± 1 °C and a relative humidity of approximately 65%.

A wide range of doses were screened for heterocyclic amine compounds, and 3 doses (4.69, 9.38 and 23.45 µM) with lower genotoxicity were used. In determining the chlorophyll pigment doses, we refer to our previous studies [17,18]. Distilled water, which has no genotoxic effect and in which the chemicals are dissolved, was used as negative control. In addition, EMS, which is known to be mutagenic, was used as a positive control to ensure that test system was working [19]. The positive control was only used to demonstrate that the test system was working and was not included in any statistical comparison. Therefore, the positive control application was not repeated at 48 ± 4 h applications. DMSO was used as a solvent control.

We used two methods to determine the antigenotoxic effect.

#### 2.3.1. MeIQx and Chlorophyll *a* and Chlorophyll *b* Co-Treatment Applications

Dose screening was performed to determine the doses to be used for MeIQx. In the 72 ± 4 h larval application, 3 doses having nontoxic but genotoxic effects were determined (4.69, 9.38 and 23.45 µM). These 72 ± 4 h larval applications were performed for distilled water, EMS, DMSO, and chlorophyll *a* and chlorophyll *b* (0.5 and 1 µM). Thus, it was determined whether these substances were genotoxic or not. Then, chlorophyll *a* and chlorophyll *b* doses exposed to 72 ± 4 h *Drosophila* larvae with genotoxic doses MeIQx, simultaneously.

#### 2.3.2. Chlorophyll *a* and Chlorophyll *b* Pre-Treatment Applications

In this group, distilled water, DMSO, chlorophyll *a* and chlorophyll *b* doses were applied to 48 ± 4 h old larvae. The second instar (48 ± 4 h) larvae were first exposed to doses of chlorophyll *a* and chlorophyll *b* (0.5, and 1 µM). After 24 h (72 ± 4 h old), the larvae were floated off with tap water and then were treated by the genotoxic doses of MeIQx (4.69, 9.38 and 23.45 µM).

### 2.4. Preparation and Microscopic Analysis of the Wings

After metamorphosis, transheterozygous flies were collected and then stored in a 70% ethanol solution at +4 °C. Afterwards, the wings, using a Nikon SMZ645 model stereo microscope, were removed and mounted on slides in Faure’s solution (30 g of gum arabic, 30 mL glycerol, 50 g chloral hydrate and 50 mL distilled water). They were scored using a Nikon YS100 model light microscope (400× magnification) for the presence of clones of cells showing malformed wing hairs. Wings were scored for (1) small (1–2 cells) single spots, (2) large (>2 cells) single spots, both with *mwh* or *flr^3^* phenotype, and (3) twin spots (phenotypes *mwh* and *flr^3^* in adjacent clone) [16].

### 2.5. Statistical Analysis

To evaluate the statistical significance of the results, we followed the multiple decision procedure of Frei and Wurgler [20], which has four different diagnoses: positive, weakly positive, negative or inconclusive. This method tests for two alternative hypotheses: (i) the mutation frequency in the treated group is not higher than the mutation frequency in the control group; and (ii) the frequency in the treated group is not less than m times as high as the observed spontaneous mutation frequency in the control. For the statistical calculations, the conditional binomial test according to Kastenbaum and Bowman [21] was used at 5% significance levels.

The percentage of inhibition was also calculated as follows, according to Abraham [22]:

(1) $$Inhibition=\frac{a-b}{a}\times 100.$$

Here, a is the frequency of total spots induced by genotoxic agents alone and b is the frequency of total spots induced by genotoxic agents in the presence of chlorophyll *a* and chlorophyll *b*.

## 3. Results

We first performed a genotoxic dose screening for HCA to determine the protective effects of chlorophyll *a* and chlorophyll *b* against the genotoxic effects of MeIQx with the *Drosophila* wing somatic mutation and recombination test and determined to use doses 4.69, 9.38 and 23.45 µM. We also investigated genotoxic effects of chlorophyll *a* and chlorophyll *b* with negative, positive, and solvent control groups. Since MeIQx and chlorophyll were dissolved in 0.5% DMSO, these compounds were compared with DMSO instead of distilled water and statistical analyses were made. Results from DMSO were compared with those of distilled water to determine the effect of the DMSO compound. The results of the control groups are given in Table 1. The spontaneous mutation frequency of distilled water was 0.87 for individuals with the *mwh/flr^3^* genotype. No genotoxic effects of DMSO, chlorophyll *a* and chlorophyll *b* were observed in 48 ± 4 and 72 ± 4 h old larvae. (Table 1).

**Figure 1:**
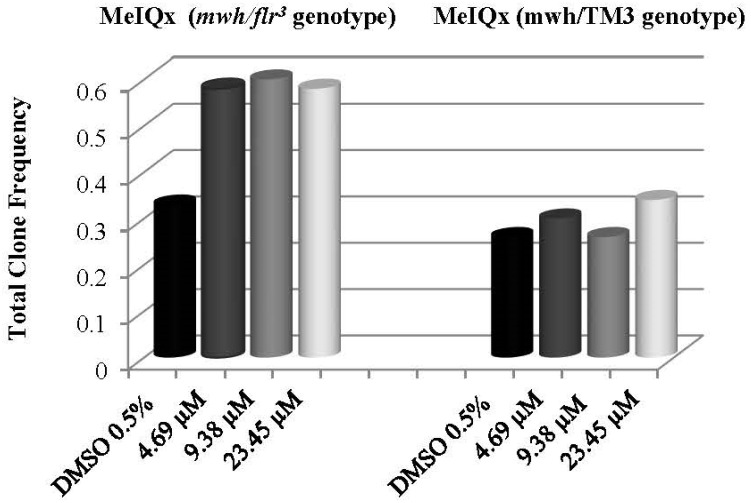
Total clone frequency distributions of MeIQx in individuals with *mwh/flr3* and mwh/TM3 genotypes.

The results obtained from 72 ± 4 h larval administration of three doses (4.69, 9.38 and 23.45 µM) determined as a result of the preliminary studies of MeIQx are given in Table 2. Genotoxic effects were observed in individuals with *mwh/flr^3^* genotype at all three concentrations. When the results were compared with the results of individuals with the *mwh*/TM3 genotype, it was observed that they showed a recombinogenic effect (Table 2). The 4.69, 9.38 and 23.45 µM MeIQx doses showed 40, 56.67 and 32% recombinogenic effects, respectively. The observed frequency increase in individuals with *mwh*/TM3 genotype was statistically insignificant. The frequency distribution in total clones is shown in Figure 1.

To determine antigenotoxic effects of chlorophyll in the pre- and co-treatment application groups, we compared their statistical analysis with the application doses of MeIQx. Pre-treatments and co-treatments with chlorophyll modified the genotoxicity of MeIQx.

**Figure 2:**
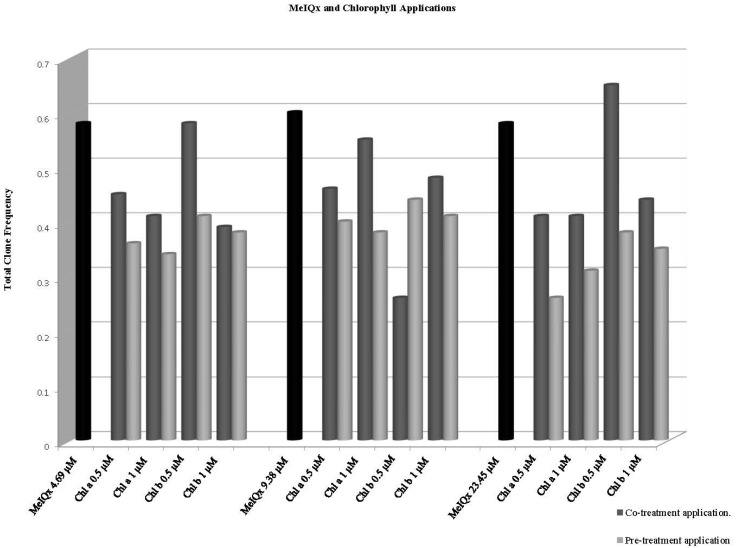
Frequency distributions of total clones as a result of chlorophyll *a* and chlorophyll *b* treatments with MeIQx.

The results of co-treatment of MeIQx with chlorophyll *a* and *b* in 72 ± 4 h larvae are given in Table 3. A decrease in total spot frequencies was observed in co-treatment applications, except that of 0.5 µM chlorophyll *b* and MeIQx 23.45 µM, which caused an increase in frequency in total spots and small single spots. In the latter case it was calculated that there was 12.06% induction in total spots, causing no statistically significant differences. As a result of other applications, chlorophyll *a* has an inhibition rate between 5.17% and 29.31%, and chlorophyll b has an inhibition rate between 0% and 55.17% in total spots. In general, co-treatment applications were found to be effective in reducing the recombinogenic effect of MeIQx (Table 3). The frequency distribution in total clones is shown in Figure 2.

The results of the pre-treatment application are given in Table 4. At all doses, chlorophyll *a* and chlorophyll *b* reduced the recombinogenic effect. Significant reductions in clone induction frequencies and percentage inhibition rates were observed. Chlorophyll *a* decreased the recombination between 31.03% and 50% and chlorophyll *b* between 24.14% and 58.62% at all MeIQx doses. The frequency distribution in total clones is shown in Figure 2.

## 4. Discussion

In this study, we investigated the antimutagenic and antirecombinogenic effects of chlorophyll *a* and chlorophyll *b* against MeIQx, which is a heterocyclic amine, by *Drosophila* wing somatic mutation and recombination test.

HCAs are food toxicants that are formed by cooking meat and meat products at high temperatures and are then ingested mostly by eating. These chemicals, which are expressed as promutagens that require metabolic activation to cause damage in DNA [25], are converted into their derivatives by being bioactivated by the cytochrome P450 enzyme system [25,26]. The rapid disappearance of radiolabeled HCAs from the environment and their unchanged form occurrence as a small amount in the urea indicate that they are metabolized to a large extent [27]. In an epidemiological study using an inhibitor of the CYP1A2 enzyme, which is a group of cytochrome P450s, the rates of MeIQx and PhIP HCAs in the urine were examined. Before the use of an inhibitory substance, HCAs were found in small amounts in the urine, while an increase in the amount of HCA excreted unchanged was observed with the administration of an inhibitor substance. As a result, it was concluded that the CYP1A2 enzyme is responsible for the metabolism of these HCAs, and their metabolism is prevented by the administration of inhibitor substances [28].

Various types of cancer, in particular colon cancer, are possible harmful effects of HCAs [27,29]. Detection of 90% of animal carcinogens as bacterial mutagens [30] has guided genotoxicological studies as well as carcinogenic studies. There are studies with various model organisms and cell groups to identify possible genetic damage that HCAs can cause [31,32,33,34,35,36,37,38]. As a result of many short-term tests, the genotoxicity of these compounds has been demonstrated, and it has been stated that they are effective mutagens in bacterial and eukaryotic cells. DNA damage accumulated in the organism may contribute to the emergence of various mutational diseases depending on the tissue and organ in which they occur [39]. Since most cancers are processes that occur as a result of various mutations at the cellular level, it is an important process to determine the methods of reducing the biological use of HCA in the organism.

Along with the discoveries of HCAs, various studies have been conducted on their formation in food, their metabolism, and their genotoxic and carcinogenic effects. Some of them aim to understand how these compounds are formed during cooking. For this purpose, the effects of different temperatures and cooking times on HCA formation have been investigated [40] by creating HCAs with modeling methods [41,42]. Furthermore, some strategies have been developed to prevent its forming during cooking [43,44]. The role of free radicals in the formation of HCA suggests that the use of antioxidant substances during cooking may be an effective method of application. For this purpose, the activities of various antioxidant substances were determined by marination method [45,46,47,48,49,50]. It has been shown that it is possible to reduce the formation of HCA with these methods, but it cannot be completely eliminated. The search for new methods and substances that will reduce the effects of these compounds after they are taken into the body continues. The chemical composition of the food is important in the formation of HCA, and the antioxidant content is essential in reducing the genotoxic effect of the HCA. In this respect, consuming different types of food at meals can be an important factor. Consumption of chemical protective factors against genetic damage that may be caused by HCAs and/or their metabolites may be important in changing the genotoxic effect.

Various studies have been conducted on the protective effects of the dietary component against the genotoxic activities of HCAs. For example, Taira et al. [51] found that two different types of edible mushrooms attenuate the DNA damage induced by MeIQx in the Drosophila DNA repair test. Takahashi et al. [52], with the same test system, determined that some anthraquinone pigments have an effective antigenotoxic effect against MeIQx.

Nakahara et al. [53] determined the flavanones isolated from the methanol extract of the flowers of *Azadirachta indica* were potent antimutagens against Trp-P-1 (3-amino-1,4-dimethyl-5H-pyrido[4,3-b]indole) in the *Salmonella typhimurium* TA98 assay. They also showed the antimutagenic effects occurred by inhibiting the enzymes responsible for the activation of heterocyclic amine compound.

Chlorophylls are the most abundant green pigments in plants and constitute the nutritional component of green fruits and vegetables. Chlorophyll and its derivatives are also used as food additives to color foods [54]. It has a cyclic tetrapyrrole structure similar to the heme group added to the globulin structure. The central metal atom is iron in the heme ring and magnesium in the chlorophyll [55].

In this study, we investigated the recombinogenic effect of MeIQx and observed the antirecombinogenic effect of chlorophyll pigments. Chlorophyll pigments reduced the genotoxic effect of MeIQx at a rate of at most 58.62%. Although chlorophyll *b* 0.5 µM dose increased the genotoxic effect of 23.45 µM MeIQx dose (12.06%) in co-treatment applications, the statistical analysis revealed that this increase is not significant. However, we examined different studies to understand the reason for the increase in this dose. The mechanisms of action that are revealed in studies with chlorophyll and chlorophyllin, a chlorophyll derivative, have been reported to be interceptor molecules that effectively reduce the HCA activity of these pigments. Arimoto et al. [56] examined the inhibition mechanisms of 3 different chlorophyllin against Trp-P-1 and Trp-P-2 heterocyclic amine compounds. Accordingly, chlorophyllin showed antimutagenic effects by capturing HCAs and forming complexes with them. Similarly, it has been shown that co-administration of IQ and chlorophyllin to rats increased unmetabolized mutagen in urine and feces. This confirmed that chlorophyll acts as an interceptor molecule [57]. When aflatoxin B1 was applied to rats fed with chlorophyllin and chlorophyll, it was observed that aflatoxin B1 excretion increased in the feces of the rats. Thus, mutagen uptake from the intestines decreased and antimutagenic effect emerged, which showed that chlorophyllin and chlorophyll acted by a similar mechanism [58].

According to the results we obtained, we think that similar chlorophyll pigments can form a complex with MeIQx and reduce the use of MeIQx, thus reducing the recombinogenic effect. In high-dose HCA applications, it can be said that chlorophyll *b* 0.5 µM is insufficient in terms of complex formation, so it shows a slight induction instead of inhibition. However, when the dose of chlorophyll *b* is increased, it is seen that the antirecombinogenic effect increases in parallel.

Negishi et al. [59] determined that co-treatment application of chlorophyll and chlorophyllin to 84 ± 4 h old larvae in the *Drosophila* SMART method reduced Trp-P-2 (3-amino-l-methyl-5A/-pyrido[4,3-ft]indole), which is a HCA, genotoxicity. They concluded that chlorophyll and chemical interaction might have increased due to the change in the pH of the digestive system in the late larval period, and therefore the protective activity might increase in parallel. Determining chlorophyll and chemical interactions in aqueous medium with spectrophotometric analyzes has further strengthened this idea [60]. Thus, these pigments reduced the usability of the chemical by showing a desmutagenic effect in co-treatment applications [60]. Similarly, Jubert et al. [61] suggested that chlorophyll *a* or chlorophyllin co-consumption may limit the bioavailability of ingested aflatoxin in humans, as they do in animal models. In our previous studies [62,63], we found that the protective effectiveness of both chlorophyll and chlorophyllin, a derivative of chlorophyll, at pre-application increased more than the co-treatment application with UVB. Similarly, it has been shown using *Drosophila* SMART that the mutagenicity of gamma radiation was reduced by the 48 ± 4 h pre-application of chlorophyll [64].

Our results indicate that the pre-treatment group is more effective than the co-treatment applications. More effective inhibition in the pre-treated groups suggests that the mutagen–chlorophyll interaction is not the only contribution to the antigenotoxic effect. In the pre-treatment group, the bond between MeIQx and DNA may be inhibited because of the possible interaction between pigments and DNA.

As a result of spectrophotometric data, it was shown that chlorophyll binds to the phosphate bone in DNA and to the N-7 G base region of the GC base pair in the major groove [65]. Thus, this ensured the protection of DNA nucleophilic areas that reactive oxygen species easily attack. Antimutagens that form complexes with DNA stabilize the DNA double strand. Free radicals formed during HCA metabolisms may be prevented from attacking DNA in this way. In addition, in a bacterial mutagenesis study, it was determined that chlorophyll derivatives are antimutagenic [66] and chlorophylls obtained from spinach water extract significantly reduce 8-hydroxy-2′-deoxyguanine (8-OHdG) damage and single strand DNA breakage [67]. De Cassia Bez et al. [68] suggest that chlorophyll *a* and *b* reduce the isochromatid type more effectively than the chromatidic type, in lymphocyte cultures from human peripheral blood. McQuistan et al. [69] assert that chlorophyll co-treatment provides a dose-responsive reduction in total DBC-DNA adducts without altering relative adduct intensities along the chromatographic profile, and chlorophyll co-treatment substantially inhibits liver tumor incidence and multiplicity on dibenzo (*def,p*) chrysene (DBC), which is a carcinogenic polycyclic aromatic hydrocarbon, doses up to their maximum-effect dose in trout.

In an epidemiological study by Cho et al. [70], it was stated that chlorophyll improves wrinkles and elasticity by increasing collagen production in light-damaged human skin and decreasing UV-induced epidermal keratinocyte damage. It has been also reported that chlorophyll extract protects and repairs light-induced cutin aging. In the study of Demir et al. [62,63], it was shown that chlorophyll and its derivative effectively reduce the genotoxic damage caused by UVB.

Thus, it is understood that chlorophyll pigment is an effective compound against the harmful effects of either physical agents or chemical agents. Our results in this study may suggest protective properties of the compounds that are rich in chlorophyll against DNA damage caused by HCAs such as MeIQx, which may possibly lead to further research.

Our study shows that the *Drosophila* wing somatic mutation and recombination test is a useful system that allows testing not only of a single substance but also of different substances at the same time. It is also seen as a suitable method for antigenotoxicity studies. Our results show that in the wing spot test, chlorophyll *a* and chlorophyll *b* pigments have primarily antirecombinagenic rather than antimutagenic activity.

## 5. Conclusions

In this study, we investigated the recombinogenic effect of MeIQx and observed the antirecombinogenic effect of chlorophyll pigments. Chlorophyll pigments reduce the genotoxic effect of MeIQx at a rate of at most 58.62%. Although chlorophyll *b* 0.5 µM dose increased the genotoxic effect of 23.45 µM MeIQx dose (12.06%) in co-treatment applications, the statistical analysis reveals that this increase is not significant. Our results show that chlorophyll pigments can form a complex with MeIQx and reduce the use of MeIQx, thus reducing the recombinogenic effect. Our results further indicate that the pre-treatment group is more effective than the co-treatment applications. More effective inhibition in the pre-treated groups suggests that the mutagen–chlorophyll interaction is not the only contribution to the antigenotoxic effect. In the pre-treatment group, the bond between MeIQx and DNA may be inhibited because of the possible interaction between pigments and DNA. We also note that the *Drosophila* wing somatic mutation and recombination test (SMART) is a useful and suitable method for antigenotoxicity studies.

## Figures and Tables

**Table 1:** Results from larval administration of control groups and chlorophyll *a* and *b*.

Doses	No. of Wings (N)	Small Single Spots (1–2 Cells) (*m* = 2)			Large Single Spots (>2 Cells) *(m* = 5)			Twin Spots (*m* = 5)			Total mwh Spots ^a^ (*m* = 2)			Total Spots (*m* = 2)			Frequency of Clone Formation per 10^5^ Cells ^b^
*mwh/flr^3^* wings		No	Fr	D	No	Fr	D	No	Fr	D	No	Fr	D	No	Fr	D	
72 ± 4 h treatments																	
Distilled water	80	16	0.20		1	0.01		0	0.00		17	0.21		17	0.21		0.87
1 mM EMS	80	163	2.04	+	89	1.11	+	32	0.40	+	273	3.41	+	284	3.55	+	13.99
%0.5 DMSO	80	23	0.29	-	3	0.04	i	0	0.00	i	26	0.33	i	26	0.33	i	1.33
Chlorophyll *a* 0.5	80	14	0.18	-	4	0.05	i	2	0.03	i	17	0.21	-	17	0.21	-	0.87
Chlorophyll *a* 1	80	20	0.25	-	2	0.03	-	3	0.04	i	25	0.31	-	25	0.31	-	1.28
Chlorophyll *b* 0.5	80	19	0.24	-	5	0.06	i	2	0.03	i	26	0.33	-	26	0.33	-	1.33
Chlorophyll *b* 1	80	23	0.29	-	3	0.03	i	0	0.00	i	27	0.34	-	27	0.34	-	1.38
48 ± 4 h treatments																	
Distilled water	80	17	0.21		0	0.00		1	0.01		18	0.23		18	0.23		0.92
%0.5 DMSO	80	22	0.26	i	1	0.01	i	0	0.00	i	22	0.26	i	23	0.29	i	1.13
Chlorophyll *a* 0.5	80	11	0.14	-	0	0.00	i	0	0.00	i	11	0.14	-	11	0.14	-	0.56
Chlorophyll *a* 1	80	10	0.13	-	2	0.03	i	0	0.00	i	12	0.15	-	12	0.15	-	0.61
Chlorophyll *b* 0.5	80	8	0.10	-	1	0.01	i	0	0.00	i	9	0.11	-	9	0.11	-	0.46
Chlorophyll *b* 1	80	11	0.14	-	1	0.01	i	0	0.00	i	12	0.15	-	12	0.15	-	0.61

No: number, Fr: frequency, D: statistical diagnosis according to Frei and Wurgler [20], +: positive, -: negative, i: inconclusive, m: multiplication factor, probability levels α = β = 0.05. ^a^ Considering *mwh* clones from single and twin spots. ^b^ Frequency of clone formation: *mwh* clones/wings/24,400 cells (without size correction) [23].

**Table 2:** Results from larval administration of MeIQx doses.

Doses	No. of Wings (N)	Small Single Spots (1–2 Cells) (*m* = 2)			Large Single Spots (>2 Cells) *(m* = 5)			Twin Spots (*m* = 5)			Total mwh Spots ^a^ *(m* = 2)			Total Spots (*m* = 2)			Frequency of Clone Formation per 10^5^ Cells ^b^	Recombination ^c^ (%)
*mwh/flr^3^* wings		No	Fr	D	No	Fr	D	No	Fr	D	No	Fr	D	No	Fr	D		
% 0.5 DMSO	80	23	0.29	-	3	0.04	i	0	0.00	i	26	0.33	i	26	0.33	i	1.33	
4.69 µM	80	40	0.43	+	5	0.05	i	1	0.01	i	46	0.50	+	46	0.58	+	2.04	40
9.38 µM	80	42	0.53	+	6	0.08	i	0	0.00	i	48	0.60	+	48	0.60	+	2.46	56,67
23.45 µM	80	41	0.39	+	4	0.04	i	1	0.01	i	46	0.50	+	46	0.58	+	2.04	32
*mwh*/TM3 wings																		
% 0.5 DMSO	80	19	0.24	-	2	0.03	-				21	0.26	-	21	0.26	-	1.08	
4.69 µM	80	23	0.29	i	1	0.01	i				24	0.30	i	24	0.30	i	1.23	
9.38 µM	80	20	0.25	i	1	0.01	i				21	0.26	i	21	0.26	-	1.08	
23.45 µM	80	26	0.33	i	1	0.01	i				27	0.34	i	27	0.34	i	1.38	

No: number, Fr: frequency, D: statistical diagnosis according to Frei and Wurgler [20], +: positive, -: negative, i: inconclusive, m: multiplication factor, probability levels α = β = 0.05. ^a^ Considering *mwh* clones from single and twin spots. ^b^ Frequency of clone formation: *mwh* clones/wings/24,400 cells (without size correction) [23]. ^c^ Recombination (%): [(frequency of mwh clones from *mwh/flr^3^* wings-frequency of mwh clones from *mwh*/TM3 wings)/frequency of mwh clones from *mwh/flr^3^* wings] × 100 [24].

**Table 3:** Inhibition by chlorophyll of MeIQx genotoxicity in *Drosophila*, co-treatment.

Doses	No. of Wings (N)	Small Single Spots (1–2 Cells) (*m* = 2)			Large Single Spots (>2 Cells) *(m* = 5)			Twin Spots (*m* = 5)			Total mwh Spots ^a^ *(m* = 2)			Total Spots (*m* = 2)			Frequency of Clone Formation per 10^5^ Cells ^b^	Percentage of Inhibition (↓) and Induction (↑) (%)
*mwh/flr^3^* wings		No	Fr	D	No	Fr	D	No	Fr	D	No	Fr	D	No	Fr	D		
%0.5 DMSO	80	23	0.29	i	3	0.04	i	0	0.00	i	26	0.33	i	26	0.33	i	1.54	
**4.69 µM**	**80**	**40**	**0.50**	**i**	**5**	**0.06**	**i**	**1**	**0.01**	**i**	**46**	**0.58**	**+**	**46**	**0.58**	**+**	**2.36**	
Chlorophyll *a* 0.5	80	32	0.40	i	3	0.04	-	0	0.00	i	35	0.44	-	35	0.44	-	1.79	↓24.14
Chlorophyll *a* 1	80	37	0.46	-	3	0.04	-	0	0.00	i	40	0.50	-	33	0.41	-	2.05	↓13.79
Chlorophyll *b* 0.5	80	42	0.53	i	4	0.05	i	0	0.00	i	45	0.56	-	46	0.58	i	2.30	0
Chlorophyll *b* 1	80	28	0.35	-	3	0.04	-	0	0.00	i	31	0.39	-	31	0.39	-	1.58	↓32.76
**9.38 µM**	**80**	**42**	**0.53**	**+**	**6**	**0.08**	**i**	**0**	**0.00**	**i**	**48**	**0.60**	**+**	**48**	**0.60**	**+**	**2.46**	
Chlorophyll *a* 0.5	80	33	0.41	-	5	0.06	-	0	0.00	i	36	0.45	-	38	0.48	-	1.84	↓17.24
Chlorophyll *a* 1	80	43	0.54	i	1	0.01	-	0	0.00	i	43	0.54	-	44	0.55	-	2.25	↓5.17
Chlorophyll *b* 0.5	80	21	0.26	-	0	0.00	-	0	0.00	i	21	0.26	-	21	0.26	-	1.07	↓55.17
Chlorophyll *b* 1	80	37	0.46	-	1	0.01	-	0	0.00	i	38	0.48	-	38	0.48	-	1.94	↓17.24
**23.45 µM**	**80**	**41**	**0.51**	**i**	**4**	**0.05**	**i**	**1**	**0.01**	**i**	**46**	**0.58**	**+**	**46**	**0.58**	**+**	**2.36**	
Chlorophyll *a* 0.5	80	25	0.31	-	7	0.09	i	1	0.01	i	33	0.41	-	33	0.41	-	1.69	↓29.31
Chlorophyll *a* 1	80	31	0.39	i	2	0.03	-	0	0.00	i	33	0.41	-	33	0.41	-	1.69	↓29.31
Chlorophyll *b* 0.5	80	50	0.63	i	1	0.01	-	1	0.01	i	52	0.65	i	52	0.65	i	2.66	↑12.06
Chlorophyll *b* 1	80	35	0.44	i	3	0.04	-	1	0.01	i	39	0.49	-	39	0.49	-	2.00	↓15.52

No: number, Fr: frequency, D: statistical diagnosis according to Frei and Wurgler [20], +: positive, -: negative, i: inconclusive, m: multiplication factor, probability levels α = β = 0.05. ^a^ Considering *mwh* clones from single and twin spots. ^b^ Frequency of clone formation: *mwh* clones/wings/24,400 cells (without size correction) [23].

**Table 4:** Inhibition by chlorophyll of MeIQx genotoxicity in *Drosophila*, pre-treatment.

Doses	No. of Wings (N)	Small Single Spots (1–2 Cells) (*m* = 2)			Large Single Spots (>2 Cells) *(m* = 5)			Twin Spots (*m* = 5)			Total mwh Spots *(m* = 2) ^a^			Total Spots (*m* = 2)			Frequency of Clone Formation per 10^5^ Cells ^b^	Percentage of Inhibition (↓) and Induction (↑) (%)
*mwh/flr^3^* wings		No	Fr	D	No	Fr	D	No	Fr	D	No	Fr	D	No	Fr	D		
% 0.5 DMSO	80	23	0.29	i	3	0.04	i	0	0.00	i	26	0.33	i	26	0.33	i	1.54	
**4.69 µM**	**80**	**40**	**0.50**	**i**	**5**	**0.06**	**i**	**1**	**0.01**	**i**	**46**	**0.58**	**+**	**46**	**0.58**	**+**	**2.36**	
Chlorophyll *a* 0.5	80	31	0.39	-	1	0.01	-	0	0.00	i	32	0.40	-	32	0.40	-	1.63	↓31.03
Chlorophyll *a* 1	80	24	0.30	-	4	0.05	-	0	0.00	i	28	0.35	-	28	0.35	-	1.43	↓39.66
Chlorophyll *b* 0.5	80	24	0.30	-	3	0.04	-	0	0.00	i	27	0.34	-	27	0.34	-	1.38	↓41.38
Chlorophyll *b* 1	80	17	0.21	-	1	0.01	-	1	0.01	i	19	0.24	-	19	0.24	-	0.97	↓58.62
**9.38 µM**	**80**	**42**	**0.53**	**+**	**6**	**0.08**	**i**	**0**	**0.00**	**i**	**48**	**0.60**	**+**	**48**	**0.60**	**+**	**2.46**	
Chlorophyll *a* 0.5	80	25	0.31	-	0	0.00	-	1	0.01	i	26	0.33	-	26	0.33	-	1.33	↓43.10
Chlorophyll *a* 1	80	22	0.28	-	1	0.01	-	0	0.00	i	23	0.29	-	23	0.29	-	1.18	↓50
Chlorophyll *b* 0.5	80	26	0.33	-	5	0.06	-	1	0.01	i	32	0.40	-	32	0.40	-	1.63	↓31.03
Chlorophyll *b* 1	80	21	0.26	-	0	0.00	-	0	0.00	i	21	0.26	-	21	0.26	-	1.07	↓55.17
**23.45 µM**	**80**	**41**	**0.51**	**i**	**4**	**0.05**	**i**	**1**	**0.01**	**i**	**46**	**0.58**	**+**	**46**	**0.58**	**+**	**2.36**	
Chlorophyll *a* 0.5	80	24	0.30	-	1	0.01	-	0	0.00	i	25	0.31	-	25	0.31	-	1.28	↓46.55
Chlorophyll *a* 1	80	24	0.30	-	1	0.01	-	0	0.00	i	25	0.31	-	25	0.31	-	1.28	↓46.55
Chlorophyll *b* 0.5	80	28	0.35	-	1	0.01	-	2	0.02	i	31	0.39	-	31	0.39	-	1.58	↓32.76
Chlorophyll *b* 1	80	33	0.41	-	1	0.01	-	1	0.01	i	35	0.44	-	35	0.44	-	1.79	↓24.14

No: number, Fr: frequency, D: statistical diagnosis according to Frei and Wurgler [20], +: positive, -: negative, i: inconclusive, m: multiplication factor, probability levels α = β = 0.05. ^a^ Considering *mwh* clones from single and twin spots. ^b^ Frequency of clone formation: *mwh* clones/wings/24,400 cells (without size correction) [23].

## Data Availability

Not applicable.

## Abbreviations

DMSO, Dimethyl sulfoxide; EMS, ethyl methane sulfonate; HCA, Heterocyclic amine; MeIQx, 2-amino-3,8-dimethylimidazo[4,5-f]quinoxaline; SMART, Somatic mutation and recombination test.
